# Out-of-body experiences associated with seizures

**DOI:** 10.3389/fnhum.2014.00065

**Published:** 2014-02-13

**Authors:** Bruce Greyson, Nathan B. Fountain, Lori L. Derr, Donna K. Broshek

**Affiliations:** ^1^Division of Perceptual Studies, Department of Psychiatry and Neurobehavioral Sciences, University of Virginia School of MedicineCharlottesville, VA, USA; ^2^F.E. Dreifuss Comprehensive Epilepsy Program, Department of Neurology, University of Virginia School of MedicineCharlottesville, VA, USA; ^3^Neurocognitive Assessment Laboratory, Department of Psychiatry and Neurobehavioral Sciences, University of Virginia School of MedicineCharlottesville, VA, USA

**Keywords:** epilepsy, seizures, out-of-body experience, autoscopy, near-death experience

## Abstract

Alterations of consciousness are critical factors in the diagnosis of epileptic seizures. With these alterations in consciousness, some persons report sensations of separating from the physical body, experiences that may in rare cases resemble spontaneous out-of-body experiences. This study was designed to identify and characterize these out-of-body-like subjective experiences associated with seizure activity. Fifty-five percent of the patients in this study recalled some subjective experience in association with their seizures. Among our sample of 100 patients, 7 reported out-of-body experiences associated with their seizures. We found no differentiating traits that were associated with patients' reports of out-of-body experiences, in terms of either demographics; medical history, including age of onset and duration of seizure disorder, and seizure frequency; seizure characteristics, including localization, lateralization, etiology, and type of seizure, and epilepsy syndrome; or ability to recall any subjective experiences associated with their seizures. Reporting out-of-body experiences in association with seizures did not affect epilepsy-related quality of life. It should be noted that even in those patients who report out-of-body experiences, such sensations are extremely rare events that do not occur routinely with their seizures. Most patients who reported out-of-body experiences described one or two experiences that occurred an indeterminate number of years ago, which precludes the possibility of associating the experience with the particular characteristics of that one seizure or with medications taken or other conditions at the time.

## Introduction

Alteration and impairment of consciousness are critical factors in the definition and diagnosis of epileptic seizures. There has been growing interest in the subjective descriptions of these consciousness alterations in patients with epilepsy as a source of data, in addition to objective observations of patients' behavior and communications and electroencephalographic (EEG) evidence of altered brain activity (Johanson et al., 2003).

A subjective feature sometimes reported in association with seizures is the sense of being outside the physical body. Devinsky et al. (1989) reported that 10 (6.3%) of 158 patients with epilepsy reported ictal or postictal “autoscopy,” a category that included both out-of-body experiences (9 cases) and seeing a visual image of one's double while one's center of consciousness remains inside the body (1 case). Recently, Hoepner et al. (2013) reported 5 patients with ictal autoscopy, 4 of whom reported out-of-body experiences, and all of whom had an epileptic focus “at the temporo-parietal junction or its neighboring regions” (p. 742). Purported out-of-body experiences have previously been associated with electrical stimulation of the angular gyrus near the right tempo-parietal junction (Blanke et al., 2002).

This study was designed to identify and characterize reports of out-of-body experience associated with seizure activity. We compared these reports of out-of-body experience with EEG evidence of seizure focus, in order to increase our understanding of the role of neurophysiological factors in such experiences; and with scores on a standardized measure of epilepsy-related quality of life.

## Materials and methods

### Participants

Patients attending the University of Virginia's F. E. Dreifuss Comprehensive Epilepsy Program were invited by their neurologists to participate in the study. After providing written informed consent, patients who agreed to participate were interviewed by one of us (Bruce Greyson or Lori L. Derr) regarding their recall of experiences associated with seizures.

We interviewed 100 patients with seizures. We excluded patients who had psychogenic seizures only, as well as those with intellectual impairment or psychotic symptoms severe enough to render their responses unreliable. Patients were obtained non-consecutively, as time constraints made it impossible to interview all patients with epilepsy; additionally, those patients who lacked the intellectual and linguistic capacity to be interviewed were not invited by their neurologist to participate in the study. The mean age of the 100 patients interviewed was 39.7 years (*SD* = 12.8), with a range from 18 to 70. The sample included 51 women and 49 men. The mean education level of the patients was 13.1 years (*SD* = 2.6), with a range from 4 to 19. The 100 patients included 84 Euro-Americans, 14 African Americans, and 2 Latino-Americans.

### Procedure

After soliciting an unstructured narrative description of subjective experiences associated with seizures, we administered to all patients interviewed, whether or not they claimed to recall any subjective experience, the Near-Death Experience (NDE) Scale, which includes a question specifically asking if they had ever felt separated from the body. We chose to use the NDE Scale specifically because it addressed out-of-body experiences, embedded in a series of questions about other unusual phenomena. Alternative instruments designed to assess alterations of consciousness, such as the Ictal Consciousness Inventory (Cavanna et al., 2008), do not address out-of-body sensations.

The NDE Scale consists of 16 multiple-choice items that address features commonly reported in NDEs, including cognitive changes, affective changes, purportedly paranormal processes including a sensation of being “out of the body,” and experiences of transcendence (Greyson, 1983). The NDE Scale has high internal consistency, split-half reliability, test-retest reliability, and correlation with other measures of NDE (Greyson, 1983). A Rasch rating-scale analysis established that the NDE Scale yields a unidimensional measure with interval-scaling properties that differentiates NDEs qualitatively and quantitatively from other responses to the threat of death (Lange et al., 2004).

Patients were also administered the Quality of Life in Epilepsy Scale (QOLIE-10), a 10-item Likert-type instrument designed to screen quality of life in persons with epilepsy (Cramer et al., 1996). The QOLIE-10 was developed as a brief instrument to assess the domains of seizure worry; emotional worry; energy/fatigue; cognition; physical and mental effects of medication; driving, social, and work limitations; and overall quality of life. Factor analysis yielded three factors labeled Epilepsy Effects (e.g., memory), Mental Health (e.g., depression), and Role Function (e.g., work limitations). The QOLIE-10 has demonstrated test-retest reliability, external criterion validity, and discriminant validity (Cramer et al., 1996).

The medical records of participants were examined for data on age of onset and duration of the seizure disorder, seizure frequency, and epilepsy etiology. EEG recordings were examined for EEG evidence of anatomic localization and lateralization of the seizure focus. The International League Against Epilepsy (ILAE) seizure type (Dreifuss et al., 1981), and the ILAE epilepsy syndrome (Commission on Classification and Terminology of the International League Against Epilepsy, 1989) were derived from the medical records.

### Data analysis

Patients were included in the out-of-body experience group (“experiencers”) if they either spontaneously described a sense of leaving the body associated with a seizure, or indicated on the NDE Scale that they had “clearly left my body and existed outside it” in association with a seizure. Those patients who did not report out of-body experiences associated with their seizures were designated as the comparison group.

We compared epilepsy clinic patients who reported out-of-body experiences associated with their seizures and a comparison group of patients who did not report out-of-body experiences on various facets of their seizures and neurological history and evaluation. We included comparisons involving neurophysiological data from the patients' clinic medical records, including their EEG recordings, to ascertain the anatomic focus of the seizure, the type of seizure, and the specific epilepsy syndrome; historical data on age of onset and duration of the seizure disorder, and maximum number of seizures per month.

## Results

### Subjective experiences associated with seizures

Of the 100 patients interviewed, 55 were able to recall some subjective experience associated with their seizures. Of those 55 patients, 29 (53%) reported that they could recall more than 10 seizure-associated subjective experiences, 23 (42%) reported between 2 and 10 seizure-associated experiences, and 3 (5%) reported that they could recall only 1 seizure-associated subjective experience. Thirty-nine of those patients (71%) reported that those experiences occurred during an aura immediately before their seizures, 30 (55%) reported experiences during their seizures, and 25 (45%) reported experiences during the postictal period immediately following their seizures. Percentages total more than 100% because some patients attributed their subjective experiences to more than one time period, and some could not determine when the experiences had occurred. For these reasons, it was not possible to distinguish precisely between aural, ictal, and postictal experiences. Most of these reports of subjective experience consisted of only brief, fragmentary sensory impressions rather than coherent narratives.

The kinds of subjective experiences patients reported were primarily changes in emotional state, cognitive changes, other consciousness alterations, sensory distortions, paresthesias, and other somatic sensations. Emotions, reported by 44 patients, included feeling scared, anxious, sad, apprehensive, threatened, and feeling pursued; less commonly, patients reported feeling euphoric or “protected.” Cognitive changes, reported by 40 patients, included déjà vu, racing thoughts, indecipherable thoughts, confusion, single words repeating in one's thoughts, and flashbacks from childhood. Other consciousness alterations, reported by 32 patients, included feeling tired, sleepy, “spacey,” dazed, fatigued, exhausted, intoxicated, feeling as if one is “falling into darkness,” and “no sense of order.”

Sensory distortions, reported by 55 patients, included seeing flashing lights, wavy lines, insects, geometric colored shapes, and kaleidoscopic vision, monochromatic vision, and seeing as if through a film; hearing music, pulsing noises, “a sound like Rice Krispies,” and hearing voices as if from far away or slowed down; smelling sulfur, burning, watermelon, ammonia, and pungent spices; and a bad taste in one's mouth. Paresthesias, reported by 23 patients, included feeling lightheaded, dizzy, tingling, electric jolts in the body, a stunned sensation like a nerve block, facial numbness, feeling “a sugar rush,” “butterflies,” waves of energy pulsing through the body, and burning sensations. Other somatic sensations, reported by 31 patients, included headache, pounding in one's head, tightness in the head, head swimming, nausea, sweatiness, warmth, coldness, palpitations, feeling pulled or twisted, feeling one's energy drained, weakness, and stomach ache.

### Features of near-death experience

The mean score of all participants on the NDE Scale was 1.72 (*SD* = 1.72), with a range from 0 to 6, with none of the 100 patients meeting the standard criterion of 7 points for NDEs. The number of patients endorsing each item on the NDE Scale is presented in Table 1. The most commonly reported features in association with a seizure were a sense of being out of the body and distortion of the sense of time. No patients endorsed a speeding up of their thoughts, a sense of revelation or sudden understanding, feeling of joy, sense of cosmic unity or oneness, increased sensory vividness, apparent extrasensory perception, or vision of deceased or religious spirits.

**Table 1:** **Frequency of affirmative responses to NDE Scale items associated with seizures (*N* = 100)**.

**NDE Scale item**	**Affirmative responses**
**COGNITIVE FEATURES**	
Time distortion	7
Life review or panoramic memory	2
Thought acceleration	0
Revelation or sudden understanding	0
**AFFECTIVE FEATURES**	
Overwhelming peace	1
Experience of brilliant light	1
Feeling of joy	0
Sense of cosmic unity or oneness	0
**PURPORTEDLY PARANORMAL FEATURES**	
Out-of-body experience	7
Vision of future events	2
Increased sensory vividness	0
Apparent extrasensory perception	0
**TRANSCENDENTAL FEATURES**	
Experience of another realm or world	3
Experience of a spiritual being or voice	2
Experience of a border or point of no return	1
Vision of deceased or religious spirits	0

### Reports of out-of-body experience

Of the 100 patients interviewed, none spontaneously reported out-of-body experiences as part of their open-ended narrative description of subjective experiences associated with seizures. However, in their subsequent responses on the NDE Scale, 7 patients reported a sensation of having left their bodies at some point during a seizure. When asked why they had not mentioned out-of-body experiences during their open-ended narratives, the patients commented either that they had forgotten about the out-of-body sensations until the interviewer mentioned them, or that they did not think that was what the interviewer had meant by “experiences associated with seizures.” Note that this figure of 7% represents a lifetime prevalence of out-of-body experiences associated with seizures, rather than the incidence of out-of-body experiences with seizures:

1. A 28-year-old female graduate student with symptomatic localization-related epilepsy due to periventricular nodular heterotopia had both complex partial and simple partial seizures. She also had Dandy-Walker malformation, Marfan syndrome, polycystic ovarian disease, and an extra vertebra. She reported 2-3 simple partial seizures a week, which she described as “the world coming in” without attenuation of consciousness. These events began at age 24 and lasted about 30 s. She reported about 1 complex partial seizure a month, which involved staring and inability to speak (i.e., she could think of words to say but could not produce meaningful speech) and inability to comprehend written language (i.e., printed words appeared as gibberish). The complex partial seizures could last up to 20 min, and were followed by postictal fatigue or confusion for up to 1–2 h. Her EEG showed independent bilateral temporal epileptiform spikes (left greater than right) and left parasagittal spikes. Her MRI showed bilateral periventricular nodular heterotopia and a Dandy-Walker malformation.
  She reported leaving her body during every complex partial seizure: while her body became immobile, she felt she was floating above it and could view her body and its surroundings from above. However, she reported a dual consciousness in which, while seeming to hover above her body, she also remained aware of bodily sensations. She reported that if someone brushed against her body, the sensation would “snap” her back into her body and end the seizure. The experience of being out of her body was unpleasant and alarming, as she feared something might happen to her body when she was not in control of it. She believed her perceptions from an out-of-body visual perspective were accurate and that her mind physically separated from her body, but she did not attribute any spiritual significance to that event, regarding it rather as “just something that happens” when her brain misfires. This was the only patient to report definitively that her out-of-body experiences commonly included verifiable perceptions, and the only patient to report that she had left her body frequently during seizures.
2. A 43-year-old unemployed man with cryptogenic localization-related epilepsy of unknown etiology had 2–3 complex partial seizures a year, starting at age 6. He was also diagnosed with bipolar affective disorder without psychosis, alcohol dependence, and cocaine abuse; and he had a chronic daily throbbing headache, occasionally with blurred vision, photophobia, and gastric distress. He reported that his seizures were often precipitated by stress, and he described their phenomenology as becoming unclear in his thoughts, followed by shaking and loss of consciousness, without tongue biting or loss of bowel or bladder control, followed by up to 2 hours of confusion. His EEG and MRI were normal.
  He reported having had two out-of-body experiences associated with seizures. The first occurred about 15 years ago: he felt that he definitely left his body and was flying, and that he encountered many people whom he had known in his childhood in another city. He had a profoundly beautiful experience in which a person he had known previously came to him “in an angel form” to show him a woman he would later marry, but whom he had not yet met at that time. Around 4–5 years ago he had a second out-of-body experience during a seizure: he again felt he left his body and was flying, but this time encountered no one. He had beautiful feelings of peace, love, and “oneness,” feeling that everything was interconnected; and he felt that his marriage, which was failing at that time, held a profound meaning of which he was previously unaware. This was the only patient to report a pleasurable out-of-body sensation or to attribute any spiritual significance to it.
3. A 26-year-old male college student with symptomatic localization-related epilepsy had a maximum of 20 intractable complex partial seizures a month with secondary generalization since age 15, for which he had undergone left frontal lobectomy at age 20. His seizures were described as 1 min of unresponsiveness with eyes deviating to the left, followed by progression to shaking of his right face and right upper extremity. Intensive telemetry video EEG showed independent left and right temporal slowing and frequent left central spikes; during 42 brief seizures consisting of the head moving forward and looking to the left with behavioral arrest, lasting for approximately 15–20 s, he had bihemispheric slowing, more prominent over the left, followed by diffuse attenuation of faster frequencies, and postictal slowing also more prominent over the left. MRI showed left frontal encephalomalacia and gliosis related to his surgery, and additional foci of encephalomalacia and gliosis within the right anterior frontal lobe and lateral aspect of the temporal lobes. PET/CT also showed postsurgical changes with encephalomalacia in the left frontal lobe with corresponding diminished FDG uptake. Single photon emission computed tomography (SPECT) showed increased radiotracer activity in the left temporal lobe. He was also diagnosed with depression, anxiety, and sleep apnea.
  He reported a recurrent but vague sense of leaving his body during seizures (“I feel like I'm seeing myself from somewhere else on occasion”) but could not elaborate on that description nor could he say how many times it had occurred.
4. A 30-year-old unemployed woman with symptomatic localization-related epilepsy due to subependymal cortical heterotopias had a maximum of 300 complex partial seizures a month since age 15, with rare secondary generalizations. Her seizures were characterized by behavioral arrest with right facial clonic jerking, aphasia, confusion, and postictal sleepiness. Prolonged video EEG monitoring showed interictal bilateral multifocal epileptiform discharges, most prominent in the left frontocentral region. Observed seizures were associated with frontally dominant generalized spike and wave discharges. An ictal SPECT showed increased uptake in the left temporal region. A brain MRI showed multiple bilateral subependymal heterotopias in the superior lateral aspect of the lateral ventricles. She also had been treated for depression and anxiety, with compulsive skin-picking.
  She reported 2–3 out-of-body experiences associated with seizures, which lasted between 10 and 20 s: she stated that she felt herself lift up and looked down at her inert body and could see and hear others; she stated that she felt weightless during this experience and found that frightening. She added: “It seems real, but I know it couldn't be; it's too far-fetched. I didn't see anything surprising. It may be my imagination telling me what I would look like.”
5. A 42-year-old unemployed man with symptomatic localization-related epilepsy secondary to traumatic brain injury at age 25 that required multiple craniotomies ultimately leading to a metal plate being surgically installed had a maximum of 5 complex partial seizures a week and frequent secondary generalized seizures. Prolonged video EEG monitoring showed interictal intermittent left temporal theta slowing, and independent, bilateral, frontotemporal spike discharges, more frequent on the left compared to the right. During the observed complex partial seizures, there were no definitive clinical lateralizing features, but electrographically there was evidence for left hemispheric onset. An interictal SPECT scan showed a small right temporal lobe in keeping with encephalomalacia. An MRI showed right frontal and temporal encephalomalacia with minimal left frontal encephalomalacia, as well as changes of left parietal cranioplasty exerting a mild mass effect on the underlying brain parenchyma. He described an aura of feeling lightheaded, followed by staring and drooling and a change in demeanor, with occasional hand automatisms, sometimes subsequently generalizing to a tonic clonic seizure.
  He reported one out-of-body experience associated with a seizure: he stated that he was awake during the seizure and watched himself going through it, falling down to one knee. He reported that he was aware of his brother entering the room and tried to tell his brother to stop him. He claimed to have dual consciousness in that he felt the bodily sensations of going through the seizure but also saw himself going through it. He added: “I don't remember any of the details of it. It's like a dream in my memory now.” When asked whether he believed he had left his body, he answered: “Well, obviously I don't think that sort of thing can really happen.”
6. A 30-year-old unemployed woman with idiopathic generalized epilepsy of unknown etiology had 1–2 catamenial absence seizures a month since age 15, characterized by eye fluttering and staring, and unresponsiveness that lasted up to 30 min. She also had myoclonic head jerking precipitated by stress and 1–2 tonic-clonic seizures a year. Intensive video EEG monitoring showed generalized fast spike and wave discharges consistent with the interictal findings seen in idiopathic generalized epilepsy, but no seizures were observed. A head CT and MRI were normal. She was also diagnosed with bipolar disorder, attention deficit hyperactivity disorder, and schizotypal personality disorder.
  She reported one out-of-body experience associated with a seizure 15 years ago: she felt herself rise 5–10 feet above her body and saw her body “folded up on itself.” She saw her sister run up to her, and then “everything went blank.” This patient also reported another out-of-body experience that was not associated with a seizure but rather during an “astral projection workshop,” which she felt was quite different from her seizure-related experience.
7. A 46-year-old female caretaker at a group home for disabled children, with cryptogenic localization-related epilepsy, had a maximum of one simple partial seizure a month since age 9, and tonic-clonic seizures without tongue biting or urinary incontinence less than once a year. Her typical seizures were characterized by an aura of déjà vu followed by staring, unresponsiveness, and rocking activity, followed by postictal confusion lasting up to an hour with body aches, headache, and fatigue. She also had depression, sleep apnea, hypothyroidism, and type 1 diabetes mellitus, and reported that her seizures seemed to be precipitated by hypoglycemia or emotional stress. She had had two head injuries, at ages 23 and 45, from falls secondary to hypoglycemia. Her EEG showed left temporal sharp waves, but her MRI and head CT were normal.
  She reported one episode of feeling as if she were looking into her head, as if she were an observer of her own thoughts. She felt at that time as if she were “floating in the universe,” seeing blackness, but with planets and stars. She reported that experience as very frightening because she had no control over the floating.

### Demographic and subjective experiential correlates of out-of-body experience

As shown in Table 2, the 7 patients who reported out-of-body experiences and the remaining 93 patients who did not were statistically comparable in terms of age, gender, education, and ethnicity.

**Table 2:** **Demographics among patients with and without out-of-body experiences (OBEs)**.

	**OBE (*N* = 7)**	**No OBE (*N* = 93)**	**Statistical test**
Mean age	34.1 (*SD* = 8.5)	40.2 (*SD* = 13.0)	*t* = 1.20, *df* = 98; NS
Gender			χ^2^ = 0.11, *df* = 1; NS
Female	4 (47%)	47 (51%)	
Male	3 (43%)	46 (49%)	
Years of education	13.7 (*SD* = 1.9)	13.0 (*SD* = 2.7)	*t* = 0.64, *df* = 98; NS
Ethnicity			χ^2^ = 0.15, *df* = 2; NS
Euro-American	6 (86%)	78 (84%)	
African-American	1 (14%)	13 (14%)	
Latino-American	0 (0%)	2 (2%)	

The frequency of recalled subjective experiences associated with seizures was statistically comparable between those patients who reported out-of-body experiences and those who did not (χ^2^ = 2.78, *df* = 3; NS). Among the 7 patients who reported out-of-body experiences associated with their seizures, 2 (29%) attributed their recalled subjective experiences to the aura prior to the seizure, 5 (71%) to the seizure itself, and 1 (14%) to the postictal period. The percent of patients who reported out-of-body experiences and of those who did not were statistically comparable for those experiences attributed to the pre-ictal aura (χ^2^ = 1.88, *df* = 1; NS), for the seizure itself (χ^2^ = 1.13, *df* = 1; NS), and for the postictal period (χ^2^ = 2.21, *df* = 1; NS).

As shown in Table 3, with the Bonferroni correction for multiple simultaneous statistical tests, those patients who reported out-of-body experiences scored higher than did other patients on the NDE Scale and on the individual items assessing out-of-body experience and a sense of being in an unearthly realm.

**Table 3:** **NDE Scale scores of patients with and without out-of-body experiences (OBEs)**.

	**OBE (*N* = 7)**	**No OBE (*N* = 93)**	**Statistical test**
NDE Scale (range = 0–32)	4.71 (*SD* = 1.70)	1.49 (*SD* = 1.50)	*t* = 5.43, *df* = 98; *p* < 0.001
**ITEM (RANGE = 0–2)**			
Time distortion	0.71 (*SD* = 0.95)	0.39 (*SD* = 0.59)	*t* = 1.35, *df* = 98; NS
Thought acceleration	0.29 (*SD* = 0.49)	0.13 (*SD* = 0.34)	*t* = 1.15, *df* = 98; NS
Life review	0.43 (*SD* = 0.79)	0.11 (*SD* = 0.35)	*t* = 2.12, *df* = 98; NS
Sudden understanding	0.00 (*SD* = 0.00)	0.03 (*SD* = 0.18)	*t* = −0.48, *df* = 98; NS
Sense of peace	0.00 (*SD* = 0.00)	0.11 (*SD* = 0.35)	*t* = −0.82, *df* = 98; NS
Feeling of joy	0.00 (*SD* = 0.00)	0.03 (*SD* = 0.18)	*t* = −0.48, *df* = 98; NS
Sense of cosmic unity	0.00 (*SD* = 0.00)	0.02 (*SD* = 0.15)	*t* = −0.39, *df* = 98; NS
Bright light	0.29 (*SD* = 0.49)	0.11 (*SD* = 0.35)	*t* = 1.28, *df* = 98; NS
Sensory vividness	0.14 (*SD* = 0.38)	0.19 (*SD* = 0.40)	*t* = −0.33, *df* = 98; NS
Extrasensory perception	0.00 (*SD* = 0.00)	0.03 (*SD* = 0.18)	*t* = −0.48, *df* = 98; NS
Precognitive vision	0.00 (*SD* = 0.00)	0.08 (*SD* = 0.34)	*t* = −0.59, *df* = 98; NS
Out-of-body experience	2.00 (*SD* = 0.00)	0.17 (*SD* = 0.38)	*t* = 12.69, *df* = 98; *p* < 0.001
Unearthly realm	0.57 (*SD* = 0.98)	0.04 (*SD* = 0.25)	*t* = 3.93, *df* = 98; *p* < 0.001
Mystical presence	0.29 (*SD* = 0.76)	0.03 (*SD* = 0.23)	*t* = 2.22, *df* = 98; NS
Visible spirits	0.00 (*SD* = 0.00)	0.01 (*SD* = 0.10)	*t* = −0.27, *df* = 98; NS
Border	0.00 (*SD* = 0.00)	0.02 (*SD* = 0.21)	*t* = −0.27, *df* = 98; NS

### Seizure history

The mean age at onset of seizures for the 97 patients for whom such data were available was 18.7 years (*SD* = 13.1), with a range from 0 to 65. The mean duration of the seizure disorder for those 97 patients was 20.9 years (*SD* = 13.5), with a range from 1 to 57 years. The mean maximum seizure frequency of the 91 patients for whom data were available was 42.0 per month (*SD* = 72.8), with a range from <1 to 300.

As shown in Table 4, patients who reported out-of-body experiences and those who did not were statistically comparable in terms of age of onset, duration of seizure disorder, and maximum seizure frequency.

**Table 4:** **Seizure history among patients with and without out-of-body experiences (OBEs)**.

	**OBE (*N* = 7)**	**No OBE (*N* = 93)**	**Statistical test**
Age of onset	15.7	18.9	*t* = 0.58, *df* = 94; NS
	(*SD* = 7.7)	(*SD* = 13.3)	
Years of seizure	19.3	21.0	*t* = 0.29, *df* = 94; NS
disorder	(*SD* = 14.0)	(*SD* = 13.5)	
Maximum seizures/month	51.3	41.2	*t* = 0.35, *df* = 89; NS
	(*SD* = 110.2)	(*SD* = 69.8)	

### Seizure characteristics

Epilepsy etiology was unknown for 58 patients. Among the remaining 42 patients, 29 (69%) had seizures related to focal pathology, including focal congenital malformation, mesial temporal sclerosis, chronic localized encephalitis, and benign tumor; and 13 (31%) to generalized or multifocal pathology, including diffuse head injury, generalized congenital malformation, perinatal anoxia, and multiple intracerebral hemorrhages.

Seizure type was classifiable for 97 patients, of whom 71 (73%) had complex partial seizures; 10 (10%) had simple partial seizures, including focal motor, somatosensory, autonomic, déjà vu, and cognitive seizures; and 16 (16%) had generalized seizures, including tonic-clonic, absence, and myoclonic seizures or multiple generalized.

Epilepsy syndrome was classifiable for 56 patients, of whom 44 (81%) had a localization-related syndrome, including mesial temporal lobe (7 patients), frontal lobe (5), parietal lobe (2), as well as non-classified cryptogenic (30); and 12 (22%) had a generalized epilepsy syndrome, including juvenile myoclonic (3 patients), and other idiopathic (7), as well as non-specific symptomatic generalized (1) and cryptogenic generalized epilepsy (1).

Epilepsy etiology, seizure type, and epilepsy syndrome are presented in Table 5, listed separately for those patients who did and did not report out-of-body experiences. None of these seizure characteristics differentiated the two groups.

**Table 5:** **Seizure characteristics among patients with and without out-of-body experiences (OBEs)**.

	**OBE (*N* = 7)**	**No OBE (*N* = 93)**	**Statistical test**
Epilepsy etiology			χ^2^ = 0.01, *df* = 2; NS
Focal pathology	2 (29%)	27 (29%)	
Generalized pathology	1 (14%)	12 (13%)	
Unknown	4 (57%)	54 (58%)	
Seizure type			χ^2^ = 0.38, *df* = 3; NS
Complex partial	5 (71%)	66 (71%)	
Simple partial	1 (14%)	9 (10%)	
Generalized	1 (14%)	15 (16%)	
Unclassified	0 (0%)	3 (3%)	
Epilepsy syndrome			χ^2^ = 0.87, *df* = 2; NS
Localization-related	6 (86%)	67 (72%)	
Generalized	1 (14%)	18 (19%)	
Unknown	0 (0%)	8 (9%)	

### EEG data

Sixty-five patients had EEG findings that included localizable epileptiform discharges, among whom 35 (57%) were localized in the temporal lobe, 6 (10%) elsewhere, and 20 (33%) were multifocal. Sixty-one patients had lateralizable epileptiform discharges, of whom 26 (40%) could be localized in the left hemisphere, 11 (17%) in the right hemisphere, 18 (28%) were bilateral, and 10 (15%) generalized. As indicated in Table 6, neither discharge localization nor lateralization significantly differentiated those patients who did and did not report out-of-body experiences.

**Table 6:** **EEG variables among patients with and without out-of-body experiences (OBEs)**.

	**OBE (*N* = 6)**	**No OBE (*N* = 80)**	**Statistical test**
Epileptiform discharge localization			χ^2^ = 1.35, *df* = 3; NS
Temporal lobe	2 (33%)	33 (41%)	
Other locus	0 (0%)	6 (8%)	
Multifocal	3 (50%)	17 (21%)	
None	1 (17%)	24 (30%)	
Epileptiform discharge lateralization			χ^2^ = 5.43, *df* = 4; NS
Left-sided	4 (67%)	22 (28%)	
Right-sided	0 (0%)	11 (14%)	
Bilateral	1 (17%)	17 (21%)	
Generalized	1 (17%)	9 (11%)	
None	0 (0%)	21 (26%)	

### Quality of life

The mean score on the QOLIE-10 for the 99 patients who were able to complete it was 25.0 (*SD* = 8.1), with a range from 12 to 44, which was not statistically different from the mean score of 25.6 (*SD* = 8.9) among a normative sample of patients with epilepsy (*t* = 0.70, *df* = 98; NS) (Bautista et al., 2007). The mean scores on the component factors were 7.1 (*SD* = 3.2) for Epilepsy Effect, 7.7 (*SD* = 2.4) for Mental Health, and 10.3 (*SD* = 4.5) for Role Function. These were statistically comparable to normative scores for patients with epilepsy for Epilepsy Effect (*t* = 1.54, *df* = 98; NS) and for Role Function (*t* = 0.78, *df* = 98; NS), but lower (reflecting better quality of life) than the mean score of 8.4 for Mental Health (*t* = 3.02, *df* = 98, *p* = 0.003) (Bautista et al., 2007).

As shown in Table 7, patients who reported out-of-body experiences and those who did not were statistically comparable in terms of overall quality of life, as well as for quality of life related to Epilepsy Effect, Mental Health, and Role Function.

**Table 7:** **Quality of life among patients with and without out-of-body experiences (OBEs)**.

	**OBE (*N* = 7)**	**No OBE (*N* = 92)**	**Statistical test**
QOLIE total score	24.2 (*SD* = 9.0)	25.1 (*SD* = 8.1)	*t* = 0.30, *df* = 97; NS
**QOLIE FACTORS**			
Epilepsy effect	7.7 (*SD* = 3.4)	7.1 (*SD* = 3.2)	*t* = 0.52, *df* = 97; NS
Mental health	7.9 (*SD* = 2.9)	7.7 (*SD* = 2.3)	*t* = 0.20, *df* = 97; NS
Role function	10.0 (*SD* = 4.4)	10.3 (*SD* = 4.5)	*t* = 0.30, *df* = 97; NS

## Discussion

### Out-of-body experiences associated with seizures

Among our sample of 100 patients, 7 reported out-of-body experiences associated with their seizures, although some of their descriptions were not definitive. This percent was comparable to the 6.3% prevalence reported by Devinsky et al. (1989), and slightly lower than the 9% prevalence of out-of-body experiences typically reported in surveys of the general population (Cardeña and Alvarado, 2013). Three of the patients in our sample reported only one out-of-body experience, 1 reported 2 such experiences, 1 reported “2 or 3” experiences, 1 reported a vague sense of leaving his body but could not estimate how many times that had happened, and 1 reported that she left her body with every seizure.

The finding that 6 of the 7 patients who reported out-of-body experiences associated with seizures described them as occurring only once or twice many years ago raises the question of whether those experiences were truly seizure-related. In view of the fact that 9% of the general population (presumably free of seizures) report out-of-body experiences once or twice in a lifetime (Cardeña and Alvarado, 2013) and the documented unreliability of patients' memories of their seizures (Heo et al., 2006; Quigg, 2011), partly as a result of anterograde amnesia from hippocampal involvement, it is conceivable that at least some of the out-of-body experiences reported in this study may not have been related to seizures but were erroneously attributed to seizures in retrospect.

Patients who reported out-of-body experiences were statistically indistinguishable from others in terms of age, gender, education, and ethnicity. They reported being able to recall subjective experiences associated with their seizures as often as did the comparison patients, and their recollections were assigned to the aura preceding the seizure, the seizure itself, and the postictal period, at the same rate as for comparison patients. Impairment of consciousness associated with seizures is central to the effect of epilepsy on quality of life, primarily due to their unpredictability (Mann and Cavanna, 2011). However, those patients who reported out-of-body experiences and those who did not were statistically comparable in terms of quality of life.

We anticipated that patients who reported out-of-body experiences would also score higher on the NDE Scale than did comparison patients, as a sense of leaving the body is one item on that scale. However, in addition to that item, patients who reported out-of-body experiences also reported with greater frequency a sense of being in some other realm or dimension. It is unclear whether that sense of being in another realm referred to the out-of-body experience itself or to a different experience, as only 1 of the 7 patients (patient # 2) included in his out-of-body experience a sense of leaving the immediate physical surroundings of the body.

### Comparison to spontaneous out-of-body experiences and induced body image distortions

Although out-of-body experiences commonly reported to occur spontaneously or in NDEs are typically pleasurable and often interpreted as spiritual experiences (Gabbard and Twemlow, 1984; Cardeña and Alvarado, 2013), only one of the patients in this sample reported his seizure-related out-of-body sensations to be pleasurable or attributed any religious or spiritual significance to the sensation of being out of the body (patient # 2). The remaining 6 felt their out-of-body experiences to be unpleasant or frightening, echoing the findings of Devinsky et al. (1989) that two of their patients found autoscopy to be the most troubling aspects of their disorder. Indeed, autoscopy associated with seizures is commonly accompanied by intense horror or fear, and may be associated with suicide (Brugger et al., 1994). Again, this negative affect contrasts with the blissful nature of the out-of-body phenomenon typically reported as part of spontaneous NDEs (Gabbard and Twemlow, 1984), and with the suicide-inhibiting effect of NDEs (Greyson, 1992).

As noted above, purported out-of-body experiences have previously been associated with electrical stimulation of the right angular gyrus near the temporo-parietal lobe (Blanke et al., 2002), and Hoepner et al. (2013) reported that out-of-body experiences were associated with seizure foci at the temporo-parietal junction “or neighboring region.” Recently, this link between the temporo-parietal junction and out-of-body sensations has been explored in persons without any known neurological dysfunction. Braithwaite et al. (2011) found that college students who reported spontaneous out-of-body experiences scored higher on a questionnaire designed to assess temporal lobe instability and disruptions in processing of body image than did students without out-of-body experiences. While that study was intriguing, the authors cautioned that questionnaires do not provide direct evidence of underlying neural function, and that if attenuated temporo-parietal discharges did occur in persons without epilepsy, the underlying neurophysiology would be unknown.

Despite these suggestive data, however, the out-of-body experiences reported by the 7 patients in this study were not associated primarily with right temporo-parietal foci. One patient had predominant left temporal discharges, 1 had left central, 1 had bilateral temporal, 1 had bilateral frontal, 1 had bilateral multifocal with left frontal predominance, and 2 had no epileptiform discharges on EEG. This is consistent with the finding of Devinsky et al. (1989) that autoscopic phenomena may be associated with a variety of seizure types, as well as with the general observation that focal seizures can affect distant and widespread regions of the brain (Bagshaw and Cavanna, 2011). Most of the research suggesting temporo-parietal correlates of out-of-body experiences has used imaging techniques that identify relatively focal activity. The development of methods for characterizing the activity of functional networks rather than discrete foci may lead to better understanding of these phenomena, particularly those associated with seizures, which can modify functional connectivity and affect resting state networks (Bagshaw and Cavanna, 2013).

Patients in the current study who did and did not report out-of-body experiences were comparable in their seizure histories, including epilepsy syndrome, epilepsy etiology, and seizure type. Complex partial seizures accounted for about 71% of the seizures in both groups. EEG findings, including lateralization and localization of abnormalities, did not differentiate those who reported out-of-body experiences from those who did not. The curious finding that the only case of déjà vu/jamais vu seizure occurred in a patient who reported an out-of-body experience may bear further investigation.

Studies of out-of-body phenomena associated with seizures have been confounded by conflicting definitions of the experience (Braithwaite et al., 2011). Hoepner et al. (2013) delineated three types of ictal autoscopic phenomena that differ in their degree of disembodiment and visual perspective: in true autoscopy, the self does not feel disembodied but remains in the physical body and visualizes a “double” in the extracorporeal space; in out-of-body experiences, the self feels fully disembodied and visualizes the physical body from an extracorporeal viewpoint; and in heautoscopy, the self feels ambiguously disembodied and the visual perspective changes between the intra- and extracorporeal. It remains an open question whether these various forms of body image distortion are related or distinct phenomena (Braithwaite et al., 2011).

Patients with epilepsy who have perceptual distortions, illusions, or hallucinations associated with their seizures generally have insight into the unreality of such perceptions, since they experience them across a spectrum of unusual experiences on many occasions associated with seizures (Bien et al., 2000; Elliott et al., 2009). In contrast, patients with thought disorders like schizophrenia generally do not have insight into the unreality of their hallucinations. If this distinction holds for out-of-body experiences, then we would expect that patients would recognize such experiences associated with seizures as hallucinatory and not real. In fact, only 1 of our 7 patients who reported out-of-body experiences (patient #1) believed that her out-of-body experiences were real. The other patients either recognized their out-of-body sensations as hallucinatory or expressed doubts, reporting, for example, “It seems real, but I know it couldn't be,” “It may be my imagination,” “It's like a dream,” or, “Obviously, I don't think that sort of thing can really happen.”

It is unclear whether a definitive belief that one had truly left the body is a distinct phenomenon or simply the extreme end of a continuum that includes more nebulous reports of out-of-body sensations that the experiencers do not believe was real. Brief survey questions cannot resolve this issue; rather, it requires detailed discussion with the experiencer, as half the people who respond affirmatively on questionnaires assessing belief in anomalous experiences do not in fact understand what they are professing but are expressing “quasi-beliefs”—propositions believed to be true without knowledge of their meaning—rather than informed beliefs, even when they are basing their knowledge on personal experiences (Jinks, 2012).

The experimental literature on *induced* out-of-body experiences has been furthermore confounded by eccentric uses of the term. Some researchers studying sensations induced by exogenous electrical stimulation included as an “out-of-body experience” any distortion of body image, regardless of whether it involved a subjective sense of leaving the physical body. Blanke et al. (2002) described as an “out-of-body experience” induced by electrical stimulation sensations of sinking into the bed, seeing one's legs become shorter or moving quickly toward one's face, and feeling that one's upper body was moving toward the legs. One study that reported “out-of-body experiences” elicited by stimulating the posterior right superior temporal gyrus acknowledged that the patient continued to perceive the environment from his real-person perspective, and not from the disembodied perspective as in spontaneous out-of-body experiences (DeRidder et al., 2007). Another reported an illusion they classified as “belonging to the class of OBEs” elicited by transcranial magnetic stimulation over the cerebellum in which the patient felt her body falling sideways out of her chair, but did not describe any visual impressions (Schutter et al., 2006). Cardeña and Marcusson-Clavertz (2012) have recently highlighted the inappropriate use of terms used to denote anomalous experiences by scholars unfamiliar with the clear and specific connotations of those terms as they are used in psychology and related disciplines. They concluded that anomalous experiences must be studied within the context of a thorough understanding of the phenomena and the correct use of terms (Cardeña and Marcusson-Clavertz, 2012).

It is unclear how comparable seizure-related autoscopy and heautoscopy or electrically induced body image distortions are to spontaneous out-of-body experiences. As noted above, the unpleasant affect associated with seizure-related out-of-body sensations is unlike the blissful sensations usually accompanying spontaneous out-of-body and NDEs. Furthermore, the sense of disembodiment induced by electrical stimulation is limited to a fixed location; experiencers perceive the environment from the visual perspective of the physical body; and experiencers perceive the event as illusory. In contrast, spontaneous out-of-body experiences often involve accurate perception of the environment (including the physical body) from an extracorporeal visual perspective; the disembodied center of consciousness may seem to move about independently of the physical body; and experiencers usually perceive the event as profoundly real (Greyson et al., 2008). Patients who report body image distortions during brain stimulation do so when their eyes are open, but not when their eyes are closed, unlike spontaneous out-of-body experiences, which typically occur with the eyes closed (Giesler-Petersen, 2008). Body image distortions elicited by brain stimulation are transitory, disappearing when the patient attempts to inspect the illusory body part, whereas spontaneous out-of-body experiences are not transitory but are maintained during examination of the body image (Neppe, 2002). Finally, somatic illusions induced by brain stimulation typically involve viewing only part of the body, usually include distortions like shortening or lengthening of limbs and movement, and are experienced as confusing; whereas spontaneous out-of-body experiences involve seeing the entire body from an extracorporeal perspective, do not include distortions, and are experienced as exceptionally lucid (Holden et al., 2006).

Whereas there is no way to establish that autoscopy and heautoscopy are anything more than illusions, it is possible to test whether subjective out-of-body experiences are more than illusions by seeking verification of the veridicality of perceptions from the extracorporeal perspective. Although people who report out-of-body experiences that occur spontaneously or in NDEs often claim to have accurate perceptions from a disembodied visual perspective (Greyson et al., 2008), only one patient in our sample (not the patient who described the sensations as pleasurable) believed unambiguously that her out-of-body perceptions were accurate perceptions of reality that could be corroborated by others. Four others described viewing events from an out-of-body perspective that they thought might or might not have been accurate, and 2 of the 7 expressed outright disbelief in the reality of their out-of-body sensations.

The term “altered state of consciousness,” which is commonly used to encompass a wide range of pathological and non-pathological conditions, including epileptic seizures and spontaneous out-of-body experiences, carries for many the implication of abnormality or dysfunction. The alternative term “non-ordinary mental expression” (NOME) has been suggested to designate anomalous experiences and related neuropsychological processes without implying pathology. Although reductionistic pathophysiological models may not encompass the entire range of such phenomena, brain areas and neurotransmitters involved in these experiences may provide a common terrain for both pathological and non-pathological NOMEs, creating a substrate for the association of phenomena such as out-of-body experiences with neuropathologic events such as epileptic seizures.

### Methodological issues

The data from this study must be interpreted with some caution. First, the small number of patients who reported out-of-body experiences reduced the likelihood of finding significant statistical differences from the patients who did not. It is possible that with a much larger sample, some of the non-significant trends noted in this study might prove to differentiate patients who report out-of-body experiences and those who do not, such as the somewhat higher incidence of left-sided and multifocal discharges among those who reported out-of-body experiences.

Another factor to be considered in evaluating the implications of this study was the role of comorbid psychiatric disorder in the association of out-of-body experiences with seizures. Sensky (1983a) noted that interest in anomalous subjective states in epilepsy was advanced by Slater and Beard (1963) and Dewhurst and Beard (1970), who specifically studied patients with comorbid psychosis and epilepsy. Of the 7 patients in our study who reported out-of-body experiences, 3 had been diagnosed with depression, 2 others with bipolar disorder, 2 with anxiety, 2 with sleep apnea, and 1 each with attention deficit hyperactivity disorder, schizotypal personality disorder, compulsive skin-picking, alcohol abuse, and cocaine abuse. Only 2 of these 7 patients who reported out-of-body experiences were not in concurrent psychiatric treatment. It is unclear whether psychiatric comorbidity may have influenced reports of out-of-body experiences in this study; that question may be a fruitful direction for future research.

As noted above, we chose to identify out-of-body experiences by administering the NDE Scale because that instrument explicit addresses the phenomenon. There are, however, other scales that may yield additional helpful information about alterations of consciousness associated with seizures. One of the most detailed measures for quantifying various aspects of consciousness, the Phenomenology of Consciousness Inventory (Pekala, 1991), has been used to examine dimensions of consciousness and its distortions during seizures (Johanson et al., 2008). However, that instrument is quite long and some of its items are complicated and difficult to understand, and some items have different meaning for patients with epilepsy than for other persons (Johanson et al., 2008, 2011); it is intended for use within 20 min of an experience (Pekala, 1991); and it does not specifically explore out-of-body experiences. The shorter and less demanding Ictal Consciousness Inventory (Cavanna et al., 2008) was specifically designed to measure level and content of consciousness during seizures, but it also does not address out-of-body experiences. It may be instructive, however, to include the Ictal Consciousness Inventory in future research on such phenomena associated with seizures. Reports of anomalous phenomena like out-of-body experiences during seizures may also be explored through unstructured interviews, such as EpiC, the Epilepsy-specific Content analysis method, although that technique is much more time-consuming and may be less practical in a clinic setting (Johanson et al., 2011).

Finally, in studying the association of out-of-body experiences with seizures, and particularly with complex partial seizures, it should be borne in mind that patients with complex partial seizures tend to have more frequent attacks, take more drugs, and suffer more adverse psychosocial stresses than patients with generalized seizures, all of which may interact to play a role in psychological symptoms (Reynolds, 1983). Additionally, it may be misleading to regard all patients with complex partial seizures as a homogenous group, as laterality and age of onset of the disorder may importantly influence psychological manifestations (Sensky, 1983b).

## Conclusion

This study elicited reports of out-of-body sensations associated with seizures in 7% of patients with epilepsy, but found no differentiating traits that were associated with patients' reports of out-of-body experiences with their seizures, either in terms of demographics, medical history including seizure risk factors and precipitants, seizure characteristics including localization and type of seizure, ability to recall subjective experiences associated with their seizures, or quality of life.

Considerable progress has been made in recent decades elucidating the neurobiologic correlates of altered states of consciousness, or NOMEs (Bagshaw and Cavanna, 2011, 2013), and specifically the role of epilepsy in elucidating the neural correlates of consciousness (Mann and Cavanna, 2011). In particular, there has been a wealth of suggestive evidence bearing on the neurological foundations of body image distortions (Blanke et al., 2002; Schutter et al., 2006; DeRidder et al., 2007). However, it may be premature to conclude from these suggestive correlations that out-of-body experiences are an epiphenomenon of particular neurophysiological conditions (Neppe, 2002; Holden et al., 2006). As noted above, the body image distortions elicited by electrical or magnetic stimulation of the brain differ phenomenologically from spontaneous out-of-body experiences in several important ways. The data from this study suggest that out-of-body experiences associated with seizures are not linked to any one region of the brain. Moreover, the findings that out-of-body experiences were reported slightly less often by patients with epilepsy than in surveys in the general population, and that patients with epilepsy who do describe out-of-body experiences report them occurring in only a small minority of their seizures, raise cautions about inferring a causal link between the seizure activity and out-of-body experiences.

## Author contributions

Bruce Greyson contributed substantially to the conception and design of this research; to the acquisition, analysis, and interpretation of data for the work; to drafting the work and revising it critically for intellectual content; gave final approval of the version to be published; and agrees to be accountable for all aspects of the work in ensuring that questions related to the accuracy or integrity of any part of the work are appropriately investigated and resolved. Nathan B. Fountain contributed substantially to the conception and design of this research; to the interpretation of data for the work; to revising the work critically for intellectual content; gave final approval of the version to be published; and agrees to be accountable for all aspects of the work in ensuring that questions related to the accuracy or integrity of any part of the work are appropriately investigated and resolved. Lori L. Derr contributed substantially to the conception and design of this research; to the acquisition of data for the work; to revising the work critically for intellectual content; gave final approval of the version to be published; and agrees to be accountable for all aspects of the work in ensuring that questions related to the accuracy or integrity of any part of the work are appropriately investigated and resolved. Donna K. Broshek contributed substantially to the conception and design of this research; to the interpretation of data for the work; to revising the work critically for intellectual content; gave final approval of the version to be published; and agrees to be accountable for all aspects of the work in ensuring that questions related to the accuracy or integrity of any part of the work are appropriately investigated and resolved.

### Conflict of interest statement

The authors declare that the research was conducted in the absence of any commercial or financial relationships that could be construed as a potential conflict of interest.
