# Corrigendum: Out-of-body experiences associated with seizures

**DOI:** 10.3389/fnhum.2014.00487

**Published:** 2014-07-10

**Authors:** Bruce Greyson

**Affiliations:** Division of Perceptual Studies, Department of Psychiatry and Neurobehavioral Sciences, University of Virginia Health SystemCharlottesville, VA, USA

**Keywords:** epilepsy, seizures, out-of-body experiences, autoscopy, near-death experience

In the Original Research Article “Out-of-body experiences associated with seizures” (Frontiers in Human Neuroscience, February 2014, Volume 8, Article 65, doi: 10:3389/fnhum.2014.00065), the Acknowledgment of funding was inadvertently deleted. We wish to acknowledge with gratitude that our work was supported by the Fundação Bial (grant number 81/08).

## Conflict of interest statement

The author declares that the research was conducted in the absence of any commercial or financial relationships that could be construed as a potential conflict of interest.

